# Life-threatening hemoperitoneum secondary to rupture of a uterine leiomyoma: A case report and review of the literature

**DOI:** 10.1016/j.ijscr.2019.07.004

**Published:** 2019-07-12

**Authors:** Adel Elkbuli, Saamia Shaikh, Mark McKenney, Dessy Boneva

**Affiliations:** a Department of Surgery, Kendall Regional Medical Center, Miami, FL, United States; b University of South Florida, Tampa, FL, United States

**Keywords:** Uterine leiomyoma, Fibroids, Hemoperitoneum, Massive transfusion protocol

## Abstract

**Introduction:**

While uterine leiomyomas are the most common pelvic tumors in females, resultant hemoperitoneum is an extremely rare and acute complication which requires emergent intervention and resuscitation. To date, less than one-hundred cases have been reported in the literature.

**Presentation of Case:**

We report a case of massive hemoperitoneum due to spontaneous rupture of a 20 cm pedunculated leiomyoma in a 74-year-old female who presented as a trauma alert. Rapidly declining hemodynamic status with an ultrasound consistent with extensive hemoperitoneum led to activation of the massive transfusion protocol and an emergent laparotomy. In the operating theater the laparotomy revealed 4 L of blood. She underwent a myomectomy and subsequently, an angiogram and embolization of a bleeding uterine artery. In all, she required transfusion of 26 units of blood. Post operatively she was discharged home on hospital day 13.

**Discussion:**

Near fatal hemoperitoneum secondary to a uterine leiomyoma may be due to traumatic or spontaneous rupture of an overlying vein or artery. Leiomyomas greater than 10 cm in size have an increased risk of rupturing. A significant amount of blood can accumulate in the peritoneum resulting in hypovolemic shock.

**Conclusion:**

Acute complications of uterine leiomyomas requiring surgical intervention are exceptionally rare. Candidates for the massive transfusion protocol must be appropriately and timely identified. Additionally, because surgery is a potential treatment for hemorrhage control in leiomyoma-related hemoperitoneum, surgeons should be aware of such complications.

## Highlights

• Hemoperitoneum secondary to venous rupture of a uterine leiomyoma is rare with less than thirty cases reported to date.
• Intraperitoneal hemorrhage caused by uterine leiomyomas can be a life-threatening condition.
• Surgical intervention is the definitive treatment and includes performing a myomectomy or hysterectomy.
• Prevention of transfusion-related complications should be a priority in candidate with Massive Transfusion Protocol.

## 1 Introduction

Uterine leiomyomas are common tumors which occur in 75% of women above the age 30 [1]. Leiomyomas are also the most common indication for hysterectomies performed in the United States [2]. These tumors can decrease one’s quality of life, carry an economic burden, and are associated with significant morbidity [3].

Hemoperitoneum secondary to rupture of a uterine leiomyoma is an extremely rare complication of this exceedingly common tumor. To date, there have been approximately 100 cases of hemoperitoneum resulting from leiomyomas reported in the literature [4,5]. Less than 30 of these cases resulted from rupture of a superficial vessel [2]. Here, we report a case of a patient who experienced life-threatening hemoperitoneum resulting from spontaneous rupture of a 20 cm pedunculated uterine leiomyoma. This case is reported with consideration to the SCARE criteria [6].

## 2 Case presentation

A 74-year-old female presented as a Level 1 Trauma Alert after sustaining a minor fall from standing. Prior to the fall, the patient complained of acute onset of abdominal pain. During the primary assessment, she was profoundly hypotensive and had an altered mental status (secondary to hemorrhagic shock). Initial hemoglobin level was 5.2. The massive transfusion protocol (MTP) was activated. Emergent bedside focused assessment with sonography for trauma (FAST) exam was positive for extensive hemoperitoneum, and the patient was immediately taken to the operating theater for an emergency laparotomy.

**Fig. 1:**
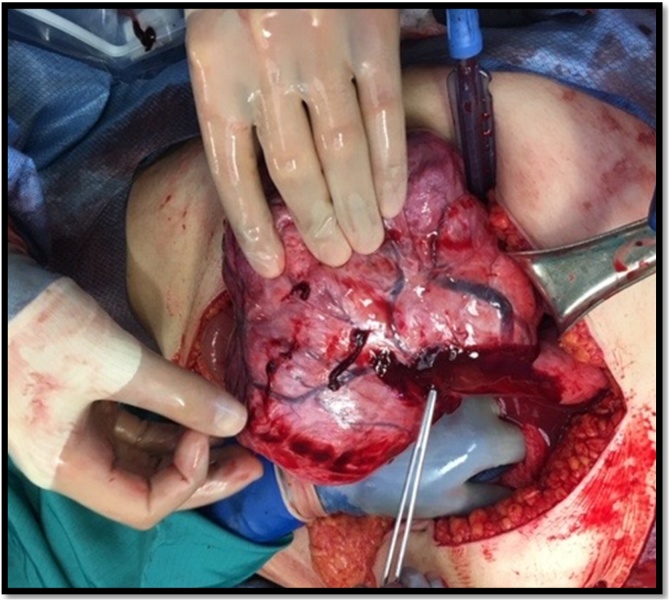
Actively hemorrhaging superficial vein overlying a pedunculated Fibroid.

**Fig. 2:**
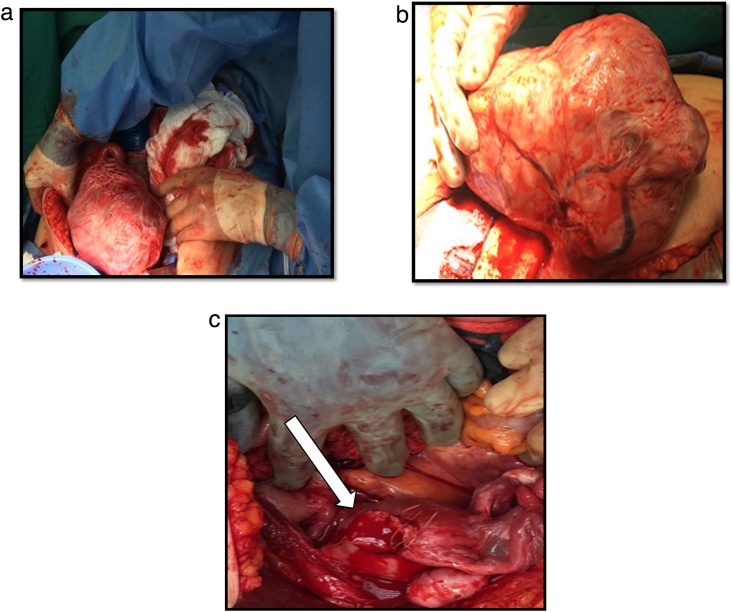
**A:** Pedunculated Fibroid seen immediately upon opening the abdomen. **B:** Pedunculated Fibroid with overlying dilated veins. **C:** Shows the uterus from top down after fibroid resection.

**Fig. 3:**
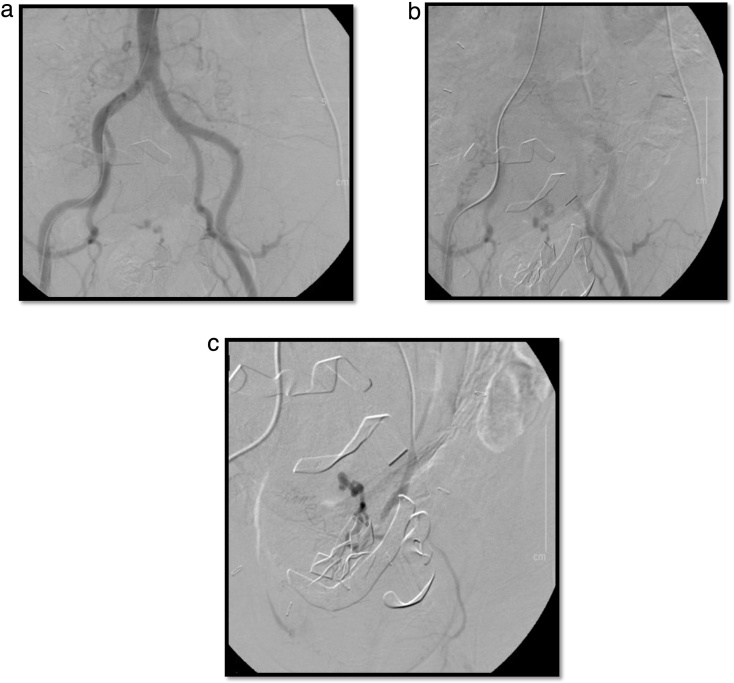
**A:** Abdominal aortic angiogram: performed via the right femoral artery. **B:** Angiogram visualizing the bilateral iliac arteries and an area with active extravasation of contrast from the left uterine artery which was subsequently embolized. **C:** Angiogram displaying successful selective gelfoam embolization of the left uterine artery. Markers from the packs which were left in the abdomen can also be seen in the image.

**Fig. 4:**
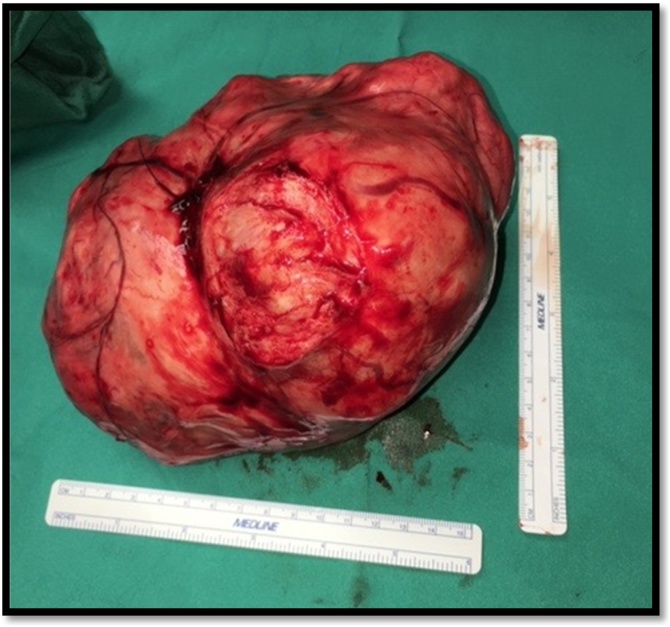
20 × 20 × 15 cm resected fibroid, gross specimen.

Upon entering the peritoneum, 4 L of blood and blood clots were encountered. On further exploration, a 20 cm pedunculated fibroid with an actively hemorrhaging superficial vein was visualized (Fig. 1). An intra-operative gynecology consultation was placed and the decision was to resect the leiomyoma with preservation of the uterus and tubes, given its prime location and visibility (Fig. 2A–C). Once control of the hemorrhaging vessel was attained, attention was turned to damage control; the abdomen was packed with combat gauze and laparotomy pads secondary to oozing from the resected tumor bed. Following surgery, the patient underwent an angiogram which revealed active extravasation of the left uterine artery. As a result, she underwent embolization of the left uterine artery (Fig. 3A–C). Post-embolization angiography was negative for any contrast extravasation. Up until this point, the patient had received a total of 26 units of blood and additional blood products (26 units of PRBC, 36 units of platelets, 21 units of FFP and 1 pooled cryoprecipitate). Final surgical pathology confirmed the diagnosis of leiomyoma and revealed focal areas with myxoid degenerative changes, the tumor measured to be 20 × 20 × 15 cm and weighed 1.25 kg (Fig. 4).

She was discharged on hospital day 13 in stable condition. Two-week follow up in the outpatient surgery clinic confirmed an uncomplicated recovery.

## 3 Discussion

Uterine leiomyomas are benign, monoclonal smooth muscle tumors and are the most common pelvic tumors in females [3]. While they may be asymptomatic, symptoms are dependent on the leiomyoma location and include fatigue, anemia, abnormal uterine bleeding, pelvic pain, urinary symptoms such as compression and frequency, and even infertility [3]. Common complications include pelvic pain arising due to degeneration, menorrhagia, and metrorrhagia [7]. Rarely, acute complications may arise and include urinary retention, renal failure, thromboembolism, and intraperitoneal hemorrhage due to torsion or avulsion of a pedunculated leiomyoma [4,8,9]. Fatal hemorrhage is an exceptionally rare complication [1].

Life-threatening hemoperitoneum related to leiomyomas may result from spontaneous or traumatic rupture of a subserosal vein or superficial dilated vein, or from a ruptured arterial aneurysm or arterial vessel arising from the uterine arteries [10]. Most cases of leiomyoma-related hemoperitoneum are due to venous rather than arterial rupture, however, and even a small venous rupture can result in a tremendous amount of intraabdominal bleeding [1,2]. Laceration, avulsion, torsion, or rupture of a degenerative fibroid or fibroid capsule have also been reported to cause hemoperitoneum [11,12]. The size of a leiomyoma is considered to be a risk factor for surface vein rupture with leiomyomas greater than 10 cm at risk. Surface vein rupture in leiomyomas ranging between 10 and 16 cm have been reported in the literature previously [10].

Additionally, vessel rupture can be either spontaneous or traumatic. Similar to the current case, reports of spontaneous venous rupture causing hemoperitoneum and hypovolemic shock have been reported [9,13,14]. Schwartz and Powell reported a case of a 53-year-old woman with a known history of uterine leiomyoma with unrelenting acute abdominal pain and a positive FAST exam. Due to a rapidly declining hemodynamic status she was rushed to the operating room. Prior to induction of anesthesia she required transfusion with a total of 10 units of blood [9]. Mizrahi et al. described a 39-year-old female with acute onset abdominal cramping and a subsequent syncopal episode. She deteriorated while in the emergency department and was immediately taken to the operating room for an emergency exploratory laparotomy with myomectomy and right uterine artery ligation. Spontaneous avulsion of a leiomyoma with active hemorrhaging from the right uterine artery was the perpetrator behind the bleeding [13]. Additionally, Gulati et al. performed a fertility-sparing myomectomy in a 29-year-old with hemoperitoneum resulting from spontaneous rupture of a serosal vessel overlying a subserosal leiomyoma [14]. In contrast to spontaneous rupture, cases with specific etiologic agents have also been reported [4]. Estrade-Huchon et al. reported life-threatening hemoperitoneum in a 46-year-old female after she had sustained a fall. Hemoperitoneum in this case resulted from traumatic avulsion of a pedunculated uterine leiomyoma [8]. Additionally, Lotterman reported hemoperitoneum in a 28-year-old female, which resulted from increased venous congestion after a bowel movement causing rupture of a surface vein [7]. Our patient experienced spontaneous venous rupture of her impressively large 20 cm leiomyoma and this coincided with the patient’s complaint of acute onset abdominal pain prior to her fall. Ultimately, accumulation of venous bleeding into the peritoneum manifested with hypotension which led to the fall.

Due to the rapidly deteriorating status of patients presenting with hemoperitoneum secondary to uterine leiomyomas, preoperative diagnosis is rare. Nonetheless, imaging modalities such as ultrasound, computed tomography scan, and magnetic resonance imaging may be used to aid with diagnosis. Additionally, in the trauma setting, imaging requires a stable patient. Therefore, while CT and MRI are able to distinguish the source of hemoperitoneum they are not useful in unstable patients. FAST exam, which is part of the standard trauma assessment, is nonspecific in that it cannot identify the source of bleeding but is still useful in that it can be quickly performed in the trauma bay and can identify intraabdominal fluid [15]. Intraabdominal blood detected by FAST coupled with a hemodynamically unstable patient is indication for emergent surgical intervention [15]. Because these patients can quickly go into hypovolemic shock due to ongoing hemorrhage, timely surgical intervention is crucial. Thus, in the trauma setting and in the case of an unstable patient, such as ours, diagnosis is often made intra-operatively [16].

Nevertheless, management of any trauma patient begins with a primary assessment (i.e., airway, breathing, circulation) and concurrent resuscitation. Management of hemorrhagic shock in a bleeding patient requires simultaneous control of the source of hemorrhage and replacement of blood volume [17]. In trauma patients, replacing blood volume loss may require activation of the MTP. Accepted definitions of massive transfusions in the literature include: (1) the transfusion of ten or more units of RBCs within 24 h [18], (2) the transfusion of more than four units of RBCs within one hour during an ongoing transfusion, and, (3) blood volume replacement of more than fifty percent of the total blood volume within three hours [19]. To minimize confusion and chaos due to the complexity of such transfusion regimens, MTPs have been developed and implemented in most trauma centers. While activating the MTP can be life-saving in select circumstances and has even been reported to be an independent predictor of survival in a subset of bleeding patients [18], it has also been associated with increased rates of mortality. Specifically, the transfusion of ten or more units of RBCs within 24 or 72 h is associated with an increased mortality [20]. Yang et al. reported that when the number of RBCs transfused in a 24-h period increases from 10 units to 40 units, an associated increase in mortality from 6.0% to 38.9% also results [20]. Como et al. demonstrated a similar relationship with mortality rates rising as high as 51% in trauma patients transfused greater than 50 units of RBCs within 24 h [21].

While supportive and resuscitative measures play a crucial role in the management of patients with massive intra-abdominal bleeding, surgery is the definitive treatment and an exploratory laparotomy should be performed immediately. Surgical approaches for bleeding leiomyomas include hemostatic suturing for primary repair, myomectomy or hysterectomy. In women of child-bearing age, preserving the uterus should be a priority. Thus, in these patients, myomectomy is the preferred surgical approach [7,16]. If bleeding cannot be controlled, however, a hysterectomy must be considered [3]. Uterine arterial embolization (UAE) is also becoming increasingly popular as it is a fertility-sparing procedure which is minimally invasive and has a shorter recovery time [22]. However, unlike elective surgical cases for leiomyomas where UAE may be the sole procedure performed, in urgent and emergent cases embolization is performed as an adjunct to surgery to obtain definite hemodynamic stability. For example, Fontarensky et al. utilized a dual treatment approach with embolization of the uterine arteries followed by hysterectomy [22]. In the current case, we sought embolization of the left uterine artery after exploration to control deep pelvic sources of bleeding.

Overall, hemorrhagic shock in a 74-year-old carries with it a considerable mortality rate. Notwithstanding, our patient did well. Resuscitation efforts initiated immediately including activation of the MTP. Expeditious and aggressive trauma resuscitation contributed to her survival. Despite requiring a massive transfusion with a total of 26 units of blood and additional blood products, our 74-year old patient was successfully extubated on hospital day four and discharged within two weeks.

Hemoperitoneum due to rupture of a vessel overlying a leiomyoma is a rare complication that presents in most commonly in women of childbearing age [2]. Thus, the presenting age of our patient of 74 years itself makes this a very unusual presentation. Additionally, we report the largest leiomyoma—20 × 20 × 15 cm—to cause hemoperitoneum and hemorrhagic shock as a result of spontaneous bleeding of a pedunculated leiomyoma. Previously in the literature, leiomyomas complicated with hemoperitoneum varied in size from 4 cm to 16.3 cm [4]. Furthermore, to our knowledge, this is the first case of uterine leiomyoma complicated with hemoperitoneum that presented as a trauma alert.

## 4 Conclusion

Acute complications of uterine leiomyomas requiring surgical intervention are exceptionally rare. Rupture of subserosal or superficial veins or arteries can result in life-threatening hemoperitoneum and hemorrhagic shock requiring immediate attention. Surgical management usually involves a myomectomy or hysterectomy and embolization of uterine arteries may play an adjuvant role.

## Conflicts of interest

None

## Funding

None.

## Ethical approval

This is a case report study. Informed written consent has been obtained and all identifying information is omitted. This work has been conducted in compliance with ethical standards.

## Consent

Informed written consent has been obtained and all identifying information is omitted.

## Author contribution

Adel Elkbuli, Saamia Shaikh, Dessy Boneva, Mark McKenney – Conception of study, acquisition of data, analysis and interpretation of data.

Adel Elkbuli, Dessy Boneva, Saamia Shaikh – Drafting the article.

Dessy Boneva, Mark McKenney – Management of case.

Adel Elkbuli, Saamia Shaikh, Dessy Boneva, Mark McKenney – Critical revision of article and final approval of the version to be submitted.

## Registration of research studies

This is a case report study.

## Guarantor

Dessy Boneva

Mark McKenney

## Provenance and peer review

Not commissioned, internally peer-reviewed.
